# Boosting PVC reactivity through iodination for CO_2_-derived functionalization

**DOI:** 10.1039/d6ra00749j

**Published:** 2026-03-06

**Authors:** Juliette Delcorps, Emna Ben Ayed, Olivier Coulembier

**Affiliations:** a Laboratory of Polymeric and Composite Materials, Centre of Innovation and Research in Materials and Polymers, University of Mons Place du Parc 23 7000 Mons Belgium olivier.coulembier@umons.ac.be

## Abstract

Aqueous phase-transfer catalysis enables selective iodination of PVC under mild and sustainable conditions. Subsequent reaction with a DBU-based CO_2_-binding amine (CO_2_BAM) achieves efficient S_N_2 substitution at CH–X centers, improving CO_2_ incorporation fourfold relative to previous CO_2_BAL strategies. The polymer remains fully soluble, demonstrating a greener, safer, and effective approach to PVC structural modification.

## Abstract 2

PVC iodination *via* phase-transfer catalysis and subsequent reaction with CO_2_BAMs opens new possibilities for simultaneous PVC and CO_2_ upcycling

## Graphical abstract

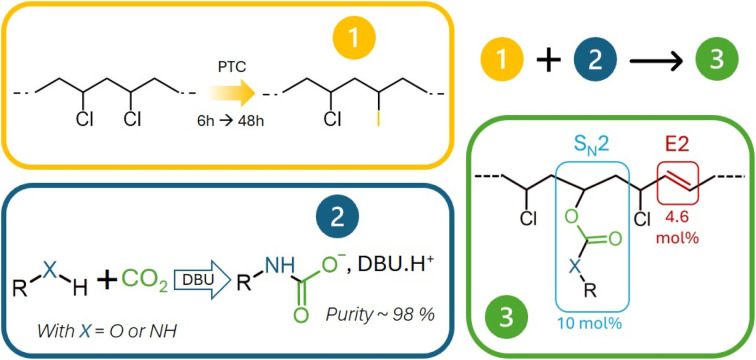

## Introduction

Among the many challenges facing sustainable materials science, the simultaneous management of carbon dioxide (CO_2_) and halogenated plastics stands out as both urgent and conceptually rich. Poly(vinyl chloride) (PVC), the third most produced polymer worldwide, combines large-scale utility with a notorious resistance to circularity.^1–5^ Its chlorine content hinders conventional recycling, while its incineration generates corrosive and toxic emissions.^6^ In parallel, the chemical community continues to seek viable strategies for CO_2_ utilization, not merely for capture, but for its integration into value-added materials.^7–13^ Bringing these two issues together, the chemical modification of PVC through CO_2_-based chemistry represents a promising step toward genuine carbon circularity.

**Fig. 1:**
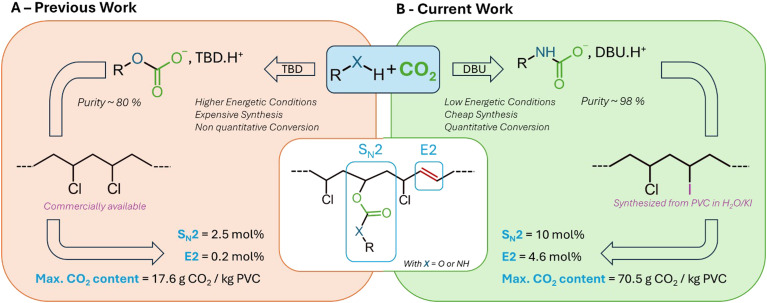
(A) Previously reported reactivity of CO_2_BALs in S_N_2 with pristine PVC. See ref. 14; (B) this work: enhancement of substitution rate *via* PVC iodination and the use of CO_2_BAMs. With TBD = 1,5,7-triazabicyclo[4.4.0]dec-5-ene, DBU = 1,8-diazabicyclo[5.4.0]undec-7-ene, S_N_2 = second-order nucleophilic substitution and E2 = second-order elimination.

Our group recently explored this concept using CO_2_-binding alcohols (CO_2_BALs), a subclass of CO_2_-binding organic liquids (CO_2_BOLs).^14^ These dual-function reagents acted both as CO_2_ carriers and as nucleophiles capable of reacting with PVC through substitution, enabling the partial incorporation of carbonate functionalities into the polymer backbone.^15^ In our previous study, the CO_2_BAL was generated from 1-decanol and 1,5,7-triazabicyclo[4.4.0]dec-5-ene (TBD) by bubbling CO_2_ at 0.2 bar and 90 °C, reaching a conversion of approximately 80%. While this approach elegantly demonstrated the feasibility of CO_2_-mediated PVC functionalization, the overall degree of incorporation remained limited to a 7 wt% mass substitution corresponding to only *ca.* 2.5 mol% of the methine sites along the chain, after 2 hours of reaction. In terms of CO_2_ loading, this corresponds to a maximum of 17.6 g per kg of PVC (Fig. 1a).

Reasoning that a greener and more efficient CO_2_-mediated functionalization of PVC would benefit from the use of safer and renewable reagents while improving reaction selectivity, we turned our attention to amine-based CO_2_-binding agents (CO_2_BAMs). In contrast to the carbonate linkages formed with CO_2_BALs, the carbamate bonds formed by CO_2_BAMs are thermodynamically more stable due to resonance delocalization between the nitrogen lone pair and the carbonyl group. This higher stability is expected to improve the durability of the grafted structure,^16,17^ its compatibility with further transformations, and ultimately the sustainability of an expected upcycling process.

In this framework, primary amines were identified as the most suitable nucleophilic partners for CO_2_ activation.^18^ Among them, 1-hexylamine was selected for its favorable green chemistry profile and potential renewability. Linear aliphatic amines such as 1-hexylamine can be synthesized from biomass-derived alcohols *via* catalytic “hydrogen-borrowing” amination.^19^ This transformation is fully atom-economic, producing only water as a by-product and aligning with principles of green chemistry. In parallel, we sought to replace the costly and moisture-sensitive TBD guanidine used in CO_2_BAL synthesis with a more affordable and robust organic base. 1,8-Diazabicyclo[5.4.0]undec-7-ene (DBU) was selected for its economic advantage (100 ml costs approximately €109, whereas 5 g of TBD amounts to €55.4) as well as for its distinct mechanistic benefits.^20,21^ As reported by McGhee *et al.*,^22^ DBU efficiently promotes carbamate anion formation and enhances S_N_2 selectivity in urethane-type bond formation under mild CO_2_ pressure, a feature recently exploited in continuous flow conditions by Bica-Schröder and co-workers.^23^

To further enhance substitution kinetics while addressing one of the major safety and sustainability issues of PVC recycling, *i.e.* the release of corrosive hydrogen chloride, we reasoned that a partial iodination of PVC prior to CO_2_BAM treatment could mitigate acid-gas formation. Although hydrogen iodide is a stronger acid than hydrogen chloride, its vapours are less corrosive toward metallic equipment and form less volatile metal halides.^24–26^ In addition, iodinated PVC displays higher S_N_2 reactivity, facilitating more efficient CO_2_ incorporation under mild conditions.

Overall, as presented herein, this dual approach, combining CO_2_BAMs with a greener preparation of iodinated PVC (Fig. 1b), provides a safer and more sustainable pathway for covalent CO_2_ upcycling into polymeric materials. Under conditions directly comparable to those previously employed for CO_2_BAL-mediated functionalization, this strategy increases the efficiency of S_N_2 substitution by roughly fourfold (10 mol% substitution rate and max. CO_2_ content ∼70.5 g per kg of polymer), yielding a polymer that remains fully soluble in organic media despite the formation of some vinyl groups *via* E2 elimination. Together, these features highlight a clear improvement in both the efficiency and versatility of CO_2_ incorporation into PVC (Table 1).

**Table 1:** Comparative overview of the CO_2_BAL and CO_2_BAM strategies under identical reaction conditions ([PVC]_0_ = [PVC-I]_0_ = 0.7 mmol L^−1^, [Cl]_0_/[CO_2_BAM or CO_2_BAL]_0_ = 1, 21 °C, 2 h, DMF)

Parameter	CO_2_BAL Strategy	CO_2_BAM Strategy
	Previous work^15^	This work
Polymer support	PVC	Partially iodinated PVC
CO_2_ carrier activation	1-Decanol + TBD	1-Hexylamine + DBU
Purity of activated species	∼80% (20 mol% free base)	∼98%
Nucleophile	Carbonate	Carbamate
Bond formed	Carbonate linkage	Carbamate linkage
S_N_2/E2 behaviour	Limited substitution	Enhanced S_N_2 selectivity
Degree of substitution	∼2.5 mol%	∼10 mol%
Max CO_2_ content	∼17.6 g per kg of PVC	∼70.5 g per kg of PVC
Solubility	Soluble	Fully soluble
Base	TBD (moisture sensitive, costly)	DBU (robust, affordable)

## Results and discussion

### PVC iodination

**Scheme 1:**
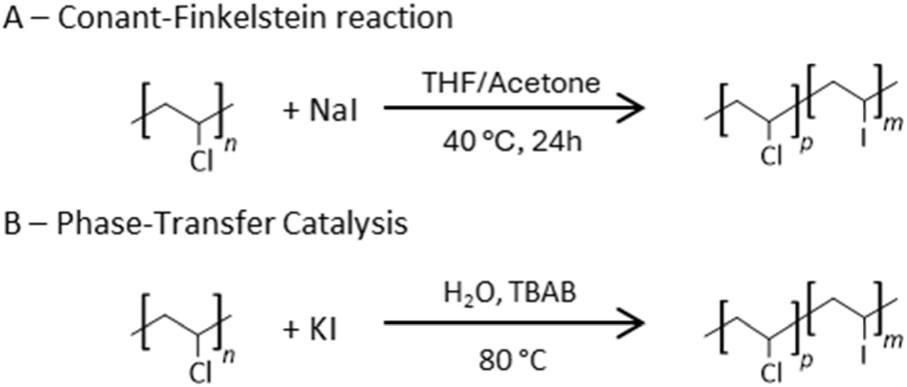
PVC iodination *via* the Conant–Finkelstein reaction (A) and phase-transfer catalysis (B). TBAB = tetrabutylammonium bromide.

In the literature, the iodination of PVC is most frequently carried out through the Conant–Finkelstein reaction using NaI in THF/acetone (Scheme 1a).^27–29^ However, this protocol relies on a problematic solvent system^30^ and does not consistently deliver high iodination efficiency. Although bio-based THF has been reported,^31–33^ replacing this mixture with safer and more sustainable media remains an important objective. An alternative route proposed by Lakshmi and Jayakrishnan uses water as solvent under phase-transfer catalysis (PTC, Scheme 1b),^34^ offering a greener and underexplored approach.

**Fig. 2:**
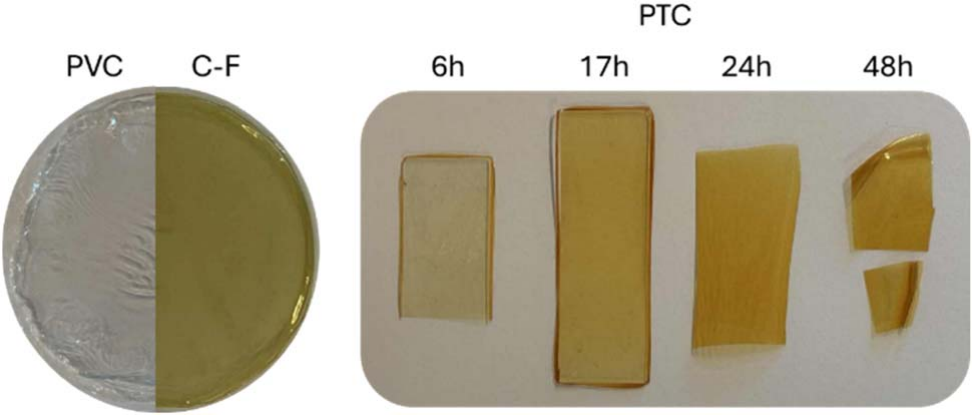
Color evolution of PVC after 24 h *via* the Conant–Finkelstein (C–F) reaction, and after 0, 6, 17, 24, and 48 h of iodination *via* PTC.

To establish a clear baseline for comparison, the conventional Conant–Finkelstein reaction was first reproduced on a smaller scale following the protocol of Rusen *et al.*^29^ This benchmark experiment not only provided direct insight into the substitution pattern and material response under classical conditions but also served as a reference for assessing the efficiency, selectivity, and environmental performance of the PTC-based alternative developed herein. After 24 hours at 40 °C, the polymer exhibited a faint yellow coloration, consistent with partial chlorine-to-iodine substitution (Fig. 2). Size exclusion chromatography (SEC) revealed a moderate increase in number-average molar mass (*M*_*n*_), while thermogravimetric analysis (TGA) showed a 32 °C decrease of the maximal degradation temperature (MDT), both supporting a PVC backbone substitution (Fig. S3 and S4). However, conventional spectroscopic techniques such as ^1^H and ^13^C NMR or FT-IR failed to provide unambiguous structural evidence, as the spectral features of PVC remain largely unaffected (Fig. S2).

To obtain quantitative insight, X-ray photoelectron spectroscopy (XPS) was employed to determine the surface iodine content, within the 1–10 nm sampling depth (Fig. S5 and S6). Although this technique does not probe the bulk of the film (*ca.* 70 µm thick), it offers a reliable benchmark for comparing different iodination methods. The XPS spectrum indicated an iodine content of 0.10 at%, confirming a moderated successful chlorine substitution ≤3% (Fig. S6 and Table S1 – entry 2). A minor sodium signal was also detected, suggesting the presence of residual NaI despite purification. Consequently, this value likely represents a slight overestimation of the true iodine incorporation. Overall, this experiment establishes the Conant–Finkelstein reaction as an effective yet limited benchmark, providing a quantitative baseline for evaluating greener iodination strategies under comparable conditions.

Building on the promising report by Lakshmi and Jayakrishnan,^34^ the phase-transfer-catalyzed (PTC) iodination of PVC was then reinvestigated. The optimized conditions reported in their original study were maintained, while reaction times were increased to enhance substitution efficiency. Reactions were thus conducted with tetrabutylammonium bromide (TBAB) used as transfer agent at 80 °C for durations of 6, 17, 24, and 48 h ([KI]_0_ = 6 M, [KI]_0_/[TBAB]_0_ = 40). After completion, the samples were thoroughly washed, dried, and solvent-casted into thin films suitable for subsequent analysis. A simple visual inspection immediately reflected the progress of iodination; while pristine PVC films remained transparent, the treated samples displayed a distinct yellow coloration that deepened with increasing reaction time (Fig. 2). Importantly, all materials remained soluble in conventional PVC solvents (THF and DMF), and none exhibited the darkening typical of extensive dehydrohalogenation. ^1^H NMR and FT-IR analyses further confirmed that elimination reactions were minimal, with the fraction of unsaturated sites remaining below 1 mol% even after 48 h (Fig. S1 and S2). This negligible degradation attests to the mildness and selectivity of the PTC approach, which proceeds efficiently under aqueous conditions without compromising polymer integrity. With the exception of the 6 h sample, all PTC-treated films exhibited a deeper yellow hue than the Conant–Finkelstein analogue, suggesting higher iodination levels. This trend was confirmed by TGA, which revealed a more pronounced MDT decrease upon increasing reaction time (Fig. S3). The relatively modest MDTs variation in TGA thermograms among the PTC samples aligns with earlier observations by Lakshmi *et al.*, who reported that substitution efficiency plateaued after prolonged exposure.^34^

SEC data showed an initial increase in *M*_*n*_ during the first 6 hours of reaction, followed by a gradual decrease at longer times (Fig. S4). Since SEC determines apparent molar masses based on hydrodynamic volume rather than absolute molecular weight, these variations reflect conformational changes in solution rather than backbone scission. Attractive intramolecular interactions are known to reduce the hydrodynamic radius of polymer chains in solution, leading to lower apparent molar masses when calibrated against polystyrene standards.^35^ Given the negligible extent of elimination, the progressive decrease in *M*_*n*_ at longer reaction times is therefore attributed to a reduction in hydrodynamic volume as the polymer backbone becomes increasingly iodinated. As the most polarizable and least electronegative halogen, iodine exhibits the strongest *σ*-hole donor character and forms particularly robust halogen bonds.^36^ Increasing iodine content thus enhances the likelihood of intra- and intermolecular halogen-bonding interactions, which promote chain compaction in solution and consequently lower the apparent *M*_*n*_.

**Fig. 3:**
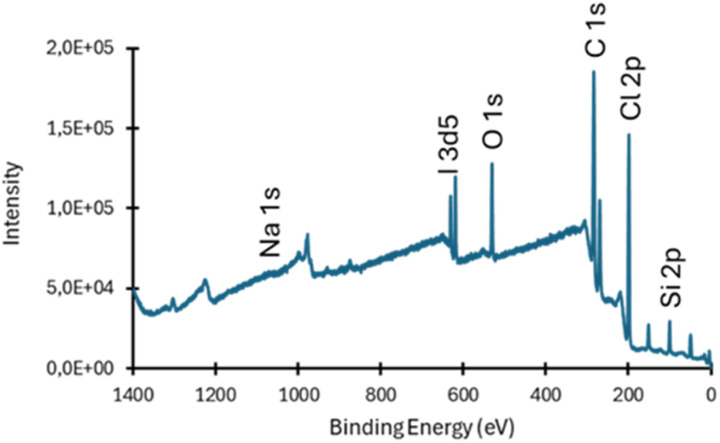
XPS spectrum of PVC iodinated *via* phase transfer catalysis (PTC) for 48 hours.

XPS measurements substantiated these findings, revealing a steady increase in iodine surface content up to 0.84 at% after 48 h, suggesting a chlorine substitution ≤24.5% (Fig. 3, S5–S9 and Table S1 – entries 3–6). In parallel, water static contact-angle measurements showed no statistically significant change between pristine PVC (85.6 ± 1.7°) and PTC-iodinated films (83.4 ± 4.3°, after 48 h), indicating that the surface wettability remains essentially unchanged despite iodine incorporation. In accord, DSC analysis showed a pronounced decrease in the glass-transition temperature, *T*_g_ (from 86 °C for pristine PVC to 50 °C after 48 h of iodination), reflecting local structural relaxation induced by the introduction of bulky iodinated groups (Fig. S10 and Table S1). Residual TBAB still detected by ^1^H NMR after purification was found to be negligible and therefore cannot account for the observed decrease in *T*_g_. Instead, this reduction is attributed to a self-plasticization effect arising from the incorporation of iodinated substituents, consistent with previous reports on bulky-substituted PVC derivatives.^37,38^

### CO_2_BAM selection and reaction with iodinated PVC

**Scheme 2:**
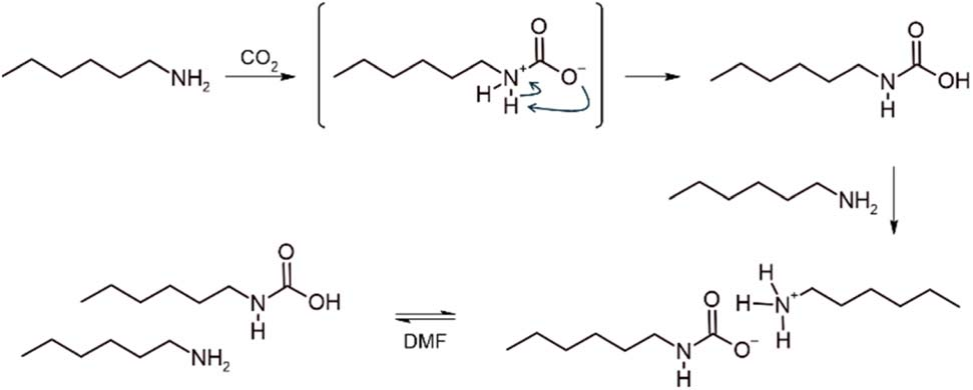
Preparation of a CO_2_BAM of 1-hexylamine through CO_2_ activation. In DMF, the carbamate form should be in equilibrium with its carbamic homologue.

Primary and secondary alkylamines react readily with CO_2_ at ambient pressure and temperature, forming carbamate or carbamic acid adducts,^18^ depending on the nature of the medium. Early reports also highlighted that the addition of organic superbases, such as amidines or guanidines, is critical for stabilizing certain amine carboxylations, enhancing both yield and nucleophilicity.^18,39,40^ Guided by this principle, three CO_2_-binding amines (CO_2_BAMs) were prepared from the representative primary amine, 1-hexylamine, for comparative evaluation. When CO_2_ was bubbled into the pristine liquid alkylamine, ^1^H NMR analysis of the medium indicated that approximately 43% of the amine reacted (Fig. S11). In this polar protic medium, and mirroring reactions performed in water,^41^ the reaction proceeds *via* formation of the carbamic acid, which is immediately deprotonated by another molecule of free amine (Scheme 2). By adding one equivalent of strong base such as 1,8-diazabicyclo[5.4.0]undec-7-ene (DBU) or 1,5,7-triazabicyclo[4.4.0]dec-5-ene (TBD), only the 1-hexylamine acts as a Lewis base while the superbase acts as Brønsted base pushing conversions in carbamate salts to 95 and 90%, respectively (Fig. S12 and S13).

Performing nucleophilic substitution directly in the bulk iodinated PVC represents the greenest approach to avoid the use of solvents entirely. Pristine PVC softens around 92 °C,^42^ whereas iodination lowers its *T*_g_ to 50–55 °C, suggesting a softening temperature near 60 °C. The three CO_2_BAMs were thus subjected to a TGA analysis to estimate their degradation onset temperatures (T5%, Fig. S15). Even the most thermally robust DBU-based CO_2_BAM (T5% = 65 °C) leaves only a narrow margin, precluding safe bulk processing, making the solvent-free strategy impractical, and the choice of a solvent necessary.

DMF was selected to ensure solubility of both iodinated PVC and carbamate salts. Although DMF is not ideal from a toxicity standpoint, the reaction conditions (see Experimental Section) produce only aqueous DMF effluents, which can be efficiently recovered *via* membrane-based techniques (pervaporation, nanofiltration), avoiding high-energy distillation or incineration.^43^ The use of DMF also informs the choice of CO_2_BAM for S_N_2 functionalization of iodinated PVC. Under a saturated CO_2_ atmosphere, the carbamate form of CO_2_BAM obtained without an organic strong base exists in equilibrium with its carbamic acid form (Scheme 2),^18^ precluding its use as a nucleophile and making it ineffective for S_N_2 substitution of the iodinated PVC. For CO_2_BAMs prepared with DBU or TBD, we previously showed that residual free base in CO_2_-binding alcohols (CO_2_BALs) promotes dehydrochlorination, forming carbon–carbon double bonds and provoking precipitation of the modified PVC. Among the CO_2_BAMs tested here, DBU provides the highest conversion in carbamate form (95%), whereas TBD reaches only 90% conversion. Considering its lower basicity (p*K*_aDBU.H^+^_ = 24.3 *vs.* p*K*_aTBD.H^+^_ = 26.0 in ACN), as well as the greater accessibility and lower cost of DBU, the DBU-based CO_2_BAM was selected as the optimal reagent for nucleophilic substitution on iodinated PVC. This choice is further supported by the excellent stability of the DBU-based CO_2_BAM in DMF over time (Fig. S14). Under identical experimental conditions as those used for the subsequent reaction with iodinated PVC ([CO_2_BAM]_0_ = 0.5 M, 21 °C, CO_2_-saturated atmosphere), the carbamate salt remained indeed fully stable for at least 24 h, with very little to no sign of CO_2_ release.

Considering the limitations of our analytical methods, it was not possible to reliably determine the iodine content of PVC-I. TGA of the iodinated material after 48 h shows an initial mass-loss event of 11–12 wt% between 90 and 140 °C, followed by a broader 53–54 wt% degradation from 140 to 400 °C (Fig. S3). For comparison, unmodified PVC typically exhibits a two-step thermal decomposition, with a major initial mass loss of ∼63 wt% and a plateau associated with char formation.^44^ This first step is often attributed to HCl elimination, but the overall mechanism is highly complex, defect-dependent, and involves overlapping processes such as dehydrohalogenation and backbone scission.^45^ In PVC-I, the splitting of the initial mass loss into two distinct events likely reflects the same intrinsic complexity, and the early 12 wt% loss cannot be unambiguously assigned to iodine elimination alone. Notably, this initial mass loss is substantially higher than the iodine surface content inferred from XPS (0.84 at%), further emphasizing the fundamentally different information depth of both techniques and precluding any direct quantitative comparison. Consequently, neither TGA, XPS nor NMR spectroscopy provides a reliable estimate of the degree of iodination. As a result, the absolute molar mass of PVC-I remains inaccessible, preventing accurate determination of a defined [halogen]_0_/[CO_2_BAM]_0_ ratio. To allow a meaningful comparison with the reactivity of pristine PVC toward CO_2_BALs reported in our previous study,^15^ we therefore elected to use the absolute number-average molar mass of unmodified PVC (*M*_*n*_,SEC = 41 400 g mol^−1^, *Đ*_*M*_ = *M*_*w*_/*M*_*n*_ = 2.2), determined in THF at 25 °C using PS calibration and Mark–Houwink corrections, as the reference value to compute the amount of CO_2_BAM. The reaction in DMF at 21 °C with the DBU-based CO_2_BAM derived from 1-hexylamine was thus conducted using this *M*_*n*_,SEC to impose an initial [halogen]_0_/[CO_2_BAM]_0_ molar ratio of 1, enabling direct comparison with the corresponding S_N_2 reactivity observed in the CO_2_BAL series. In our previous study, we demonstrated that the degree of carbonatation reached 2.5 mol% after 2 hours at 21 °C.^15^ As a proof-of-concept of our new strategy, the reaction between PVC-I and the DBU-based CO_2_BAM was then reiterated in DMF for 2 hours only ([PVC-I]_0_ = 0.7 mmol L^−1^). The reaction medium was subsequently precipitated in cold water, washed, and dried to ensure complete removal of CO_2_BAM derivatives. The structural changes induced by the reaction between PVC-I and the DBU-based CO_2_BAM were first evaluated by FTIR spectroscopy (Fig. S16). The appearance of a weak band at 1724 cm^−1^ is consistent with the presence of carbonyl groups, in line with the partial formation of carbamate-type motifs upon nucleophilic substitution. Concomitantly, a distinct absorption at 1643 cm^−1^ clearly evidences the formation of C=C double bonds, confirming that elimination processes also occur under these conditions. Additional features, namely the irregular envelope between 2000–2400 cm^−1^ and the broad, intense band between 3100–3600 cm^−1^, are characteristic of overlapping O–H, N–H and weak overtone/combination contributions.

**Fig. 4:**
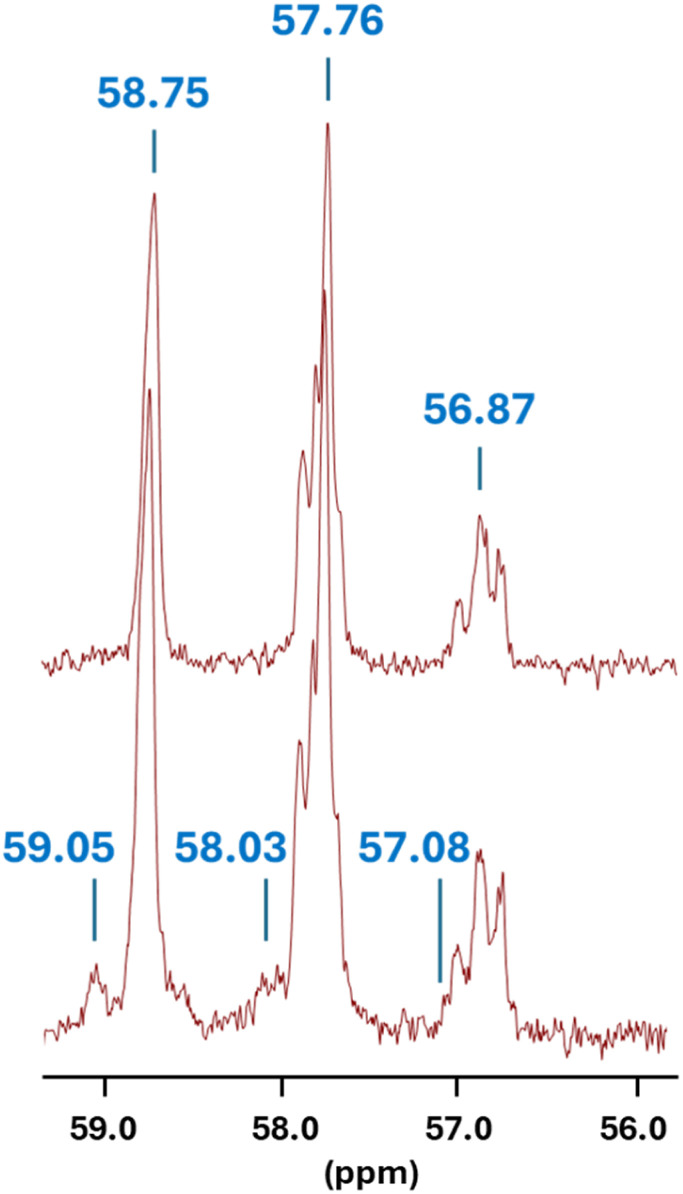
Enlarged ^13^C NMR region (*δ* = 56.0–59.2 ppm). Top: PVC-I after 48 h iodination, showing the characteristic CH–X triplet. Bottom: same region after reaction with the DBU-based CO_2_BAM of 1-hexylamine (2 h, 21 °C). Spectra recorded in THF-d_8_ at 21 °C (126 MHz).

More detailed insight was obtained from the ^13^C NMR spectrum of the modified polymer (Fig. 4). While the characteristic triplet-type CH–X pattern of PVC (*δ* = 56.87, 57.76 and 58.75 ppm, with X = Cl or I) is preserved, three new resonances emerge at *δ* = 57.08, 58.03 and 59.05 ppm. Their chemical-shift proximity and analogous triplet-like distribution strongly suggest that the modification remains localized at the CH–X carbons of the polymer backbone. The slight downfield displacement of these new signals (∼0.2–0.3 ppm) is fully consistent with partial nucleophilic substitution by CO_2_BAM-derived species or by hexylamine liberated *in situ*, although the relative contributions of carbamate retention and CO_2_ loss cannot be unambiguously established at this stage. Nevertheless, the appearance of these carbon resonances provides direct spectroscopic evidence for covalent grafting at the CH–X positions, supporting the occurrence of S_N_2-type substitution in competition with elimination.

The ^1^H NMR spectrum corroborates this mixed reactivity (Fig. S17). Integration of the vinylic protons (*δ* = 5.5–6.0 ppm) indicates 4.6 mol% of unsaturation, while signals attributable to the grafted hexyl group (*δ* = 0.7–1.5 ppm) correspond to 10.1 mol% of covalent incorporation. Taken together, these spectroscopic data depict a reaction manifold in which CO_2_BAM-derived nucleophiles engage in efficient substitution at the CH–X centers of PVC-I while a concomitant fraction of sites undergo base-promoted elimination, ultimately yielding a functionalized material with dual substitution–elimination character. Importantly, despite the formation of unsaturations, the polymer remained fully soluble throughout the reaction, in contrast to our previous observations with TBD-derived CO_2_BALs.^15^ This preserved solubility strongly suggests that the substantially higher degree of substitution achieved under CO_2_BAM/DBU conditions effectively outcompetes the elimination-inducing pathways observed in our earlier study.

## Conclusions

We report a sustainable, dual-strategy approach for covalent CO_2_ incorporation into PVC. Aqueous PTC-mediated iodination enhances S_N_2 reactivity, while DBU-based CO_2_BAMs provide stable, efficient nucleophiles for functionalization. This method increases substitution efficiency compared to CO_2_BALs, preserves polymer solubility and highlights an efficient and greener pathway for PVC chemical modification. Systematic studies on different CO_2_BAM alkyl types and reaction times are currently underway, and a comparative evaluation of the resulting polymer properties will be presented in a forthcoming paper.

## Experimental

### Materials

Poly(vinyl chloride) (low molecular weight, *M*_*n*_th = 48 kg mol^−1^, *M*_*n*_SEC = 41.4 kg mol^−1^),^15^ tetrabutylammonium bromide (TBAB), and *N*,*N*-dimethylformamide (anhydrous, 99.8%) were purchased from Sigma-Aldrich. Potassium iodide (KI), acetone p., and methanol p. were supplied by Chemlab. Sodium iodide (NaI) as well as deuterated tetrahydrofuran (99.5% D) were acquired from VWR. 1-hexylamine (99%) and 1,8-diazabicyclo[5.4.0]undec-7-ene (98+%) were supplied by Thermo Scientific. Finally, carbon dioxide N50 (≥99.999%), 1,5,7-triazabicycl[4.4.0]dec-5-ene, and deuterated chloroform (99.8%*D* + 0.03% TMS V/V, for NMR spectroscopy) were purchased from Air Liquide, Apollo Scientific, and Eurisotop, respectively.

### Experimental procedures

#### PVC iodination *via* phase transfer catalysis (PTC)

In a 100 ml single-neck round-bottom flask equipped with a stir bar were introduced 600 mg (1.25 × 10^−5^ mol) of PVC, 30 g (0.18 mol) of KI, 1.441 g (0.0045 mol) of TBAB, and 30 ml of demineralized water. The reaction medium was stirred at 80 °C for 6, 17, 24 or 48 hours. After the allotted reaction time, the reaction mixture is filtered onto a Büchner funnel and thoroughly washed with water. In order to eliminate as much unreacted TBAB and KI as possible, the sample is then solubilized in THF or MEK and reprecipitated in water.

#### PVC iodination *via* the Conant–Finkelstein reaction

In a 50 ml round-bottom flask equipped with a stir bar were introduced 1 g (2.1 × 10^−5^ mol) of PVC, 2.4 g (0.016 mol) of NaI, and 12 ml of a THF/acetone (1 : 2) mixture. The reaction mixture was then stirred under nitrogen at 40 °C for 24 hours. Once the 24 hours were up, the reaction medium was precipitated in a methanol/water (2 : 1) mixture. The precipitate was then dried overnight under vacuum at 40 °C.

#### CO_2_BAM synthesis

A Schlenk tube, equipped with a septum and a stir bar, is charged with a mixture of hexylamine (0.010 mol) and a base (0.010 mol DBU, TBD, or hexylamine itself) in bulk. A butyl pipe is used to link the Schlenk tube to a Schlenk line. This Schlenk line is used to first put the reaction mixture under vacuum, then under a carbon dioxide atmosphere (*p*CO_2_ = 0.2 bar). In the case of the DBU-based and amine only CO_2_BAMs, the reaction starts at room temperature until the exothermicity subsides (about 15 to 20 minutes). The conversion is then maximized at 90 °C in an oil bath and under maximum stirring for 30 minutes. In the case of the TBD-based CO_2_BAM, the reaction is performed directly at 90 °C for 1 hour in order to solubilize the TBD in hexylamine.

#### Substitution reaction between iodinated PVC and CO_2_BAM

Iodinated PVC sample (0.010 mol Cl, 0.6245 g PVC) was dissolved in DMF (20 ml). The CO_2_BAM (0.010 mol) was then added to the solution. The reaction was performed at room temperature, under maximum stirring, and CO_2_ atmosphere for 2 hours. After the reaction, the product is purified through a three step process. First, water is used to precipitate and wash the product. Then, the precipitate is solubilized in THF. Finally the solution from the previous step is precipitated and washed with methanol.

## Analytical methods

### Thermogravimetric analysis (TGA)

The CO_2_BAMs were subjected to thermogravimetric analysis to determine their degradation temperature. Measurements were performed under nitrogen flow at a rate of 5 °C per minute using a TA Q500 TGA. All PVC-based samples were subjected to TGA as well. Measurements were performed under nitrogen flow at a rate of 2.5 °C per minute using a TA Q500 TGA.

### Fourier transform infraRed (FT-IR) spectroscopy

FT-IR spectroscopy was performed using a Bruker Tensor 27 spectrometer to give an indication regarding the success of the substitution reaction between PVC and CO_2_BAM.

### Nuclear magnetic resonance (NMR) spectroscopy

CO_2_ conversion was followed time-to time through ^1^H NMR using a Spinsolve 60 Ultrabenchtop NMR spectrometer (Magritek, Aachen, Germany). All spectra were acquired according to the following parameters: *a*_q_ = 6.4 s, repetition time = 10 s, pulse angle = 90, *n*_s_ = 32. Final CO_2_BAM and substitution products were all characterized by ^1^H NMR using a Bruker AVANCEII 500 MHz apparatus at room temperature in chloroform-d1 (CDCl_3_) and THF-d8, respectively. All spectra were acquired according to the following parameters: *a*_q_ = 2.75 s, *d*1 = 1 s, *s*_w_ = 20, *n*_s_ = 32 for small molecules and 512 for PVC-based compounds. ^13^C NMR spectra were also acquired using the a Bruker AVANCEII 500 MHz apparatus at room temperature in THF-d8 according to the following parameters: *a*_q_ = 1.1 s, *d*_1_ = 2 s, *s*_w_ = 240, *n*_s_ = 4096.

### Size exclusion chromatography (SEC)

All PVC-based samples were subjected to SEC in THF at 25 °C using a Triple Detection Polymer Laboratories liquid chromatograph equipped with a refractive index (ERMA 7517), a UV detector (254 nm), a capillary viscometer, a light scattering RALS (Viscotek T-60) (Polymer Laboratories GPC-RI/CV/RALS), an automatic injector (Polymer Laboratories GPC-RI/UV), and four columns: a PL gel 10 µm guard column and three PL gel mixed-B 10 µm columns (linear columns for separation of *M*_*w*_ PS ranging from 500 to 10^6^ daltons). All samples for SEC analyses were prepared according to the following concentration: 1 mg of polymer sample in 1 mL of THF.

### Differential scanning calorimetry (DSC)

DSC analysis was conducted using a DSC Q2000 from TA instruments under inert atmosphere (N_2_/50 ml min^−1^). Samples of 3–4 mg were sealed in aluminum DSC pans, placed in the DSC cell and heated from −20 to 80 or 100 °C with a heating rate of 10 °C min^−1^. The glass transition temperature (*T*_g_) was measured on the second heating scan.

### X-ray photoelectron spectroscopy (XPS)

The sample composition was evaluated using XPS with a VERSAPROBE PHI 5000 instrument from Physical Electronics. The instrument was equipped with a monochromatic Al Kα X-ray source. X-ray photoelectron spectra were collected at a takeoff angle of 45°, and the electron energy analyzer was operated in the CAE (constant analyser energy) mode. To compensate for the built-up charge on the sample surface during measurements, a dual beam charge neutralization system consisting of an electron gun (approximately 1 eV) and an Ar ion gun (less than or equal to 10 eV) was employed. The binding energies were referenced to the C1s peak at 284.6 eV. Atomic concentration percentages were determined using CasaXPS software based on the XPS data.

### Contact angle

To perform contact angle measurements, films were made out of each sample by solvent casting. These films were casted directly onto microscope slides for easier handling. These measurements were performed using a Krüss Drop Shape Analysis System DSA 10 Mk2. 20 µl droplets of demineralized water were deposited in various places on the polymer film and contact angles were measured using the Drop Shape Analysis software.

## Author contributions

conceptualization: O. C.; methodology: J. D.; validation: J. D. and E. B. A.; investigation: J. D. and E. B. A.; writing original draft: J. D.; writing– review & editing: O. C., J. D., and E. B. A.; visualization: J. D., E. B. A., and O. C.; supervision: O. C.

## Conflicts of interest

There are no conflicts to declare.

## Supplementary Material

RA-016-D6RA00749J-s001

## Data Availability

The data supporting this study, including spectroscopic, thermal, and chromatographic analyses, are available in the supplementary information (SI) of this article. No additional datasets were generated or analyzed beyond those included in the main text and SI. Supplementary information is available. See DOI: https://doi.org/10.1039/d6ra00749j.
