# Supplementary material for: Boosting PVC reactivity through iodination for CO2-derived functionalization
Source: RSC Adv. 2026 Mar 6;16(14):12798–805. doi: 10.1039/d6ra00749j (PMC12965211; doi:10.1039/d6ra00749j)
Supplement: RA-016-D6RA00749J-s001 [file RA-016-D6RA00749J-s001.pdf]

Supplementary Information (SI) for RSC Advances.
This journal is © The Royal Society of Chemistry 2026

Supplementary information

Boosting PVC Reactivity through Iodination for CO_2-Derived Functionalization

Juliette Delcorps, Emna Ben Ayed, Olivier Coulembier

Laboratory of Polymeric and Composite Materials, Centre of Innovation and Research in Materials and Polymers, University of Mons, Place du Parc 23, 7000 Mons, Belgium.

E-mail: olivier.coulembier@umons.ac.be

Table des matières

1. PVC iodination....3

a. Nuclear Magnetic Resonance....3

b. Fourier Transform Infrared Spectroscopy....3

c. Thermogravimetric Analysis....4

d. Size Exclusion Chromatography....4

e. X-ray Photoelectron Spectroscopy....5

f. Differential Scanning Calorimetry....6

g. Supplementary Table S1 – Summary of physicochemical and thermal properties of pristine and iodinated PVC samples.....7

2. CO_2BAM characterization....7

a. Nuclear Magnetic Resonance....7

b. Thermogravimetric Analysis....9

3. Reaction between iodinated PVC and DBU-based CO_2BAM – Product characterization....10

1. PVC iodination

a. Nuclear Magnetic Resonance

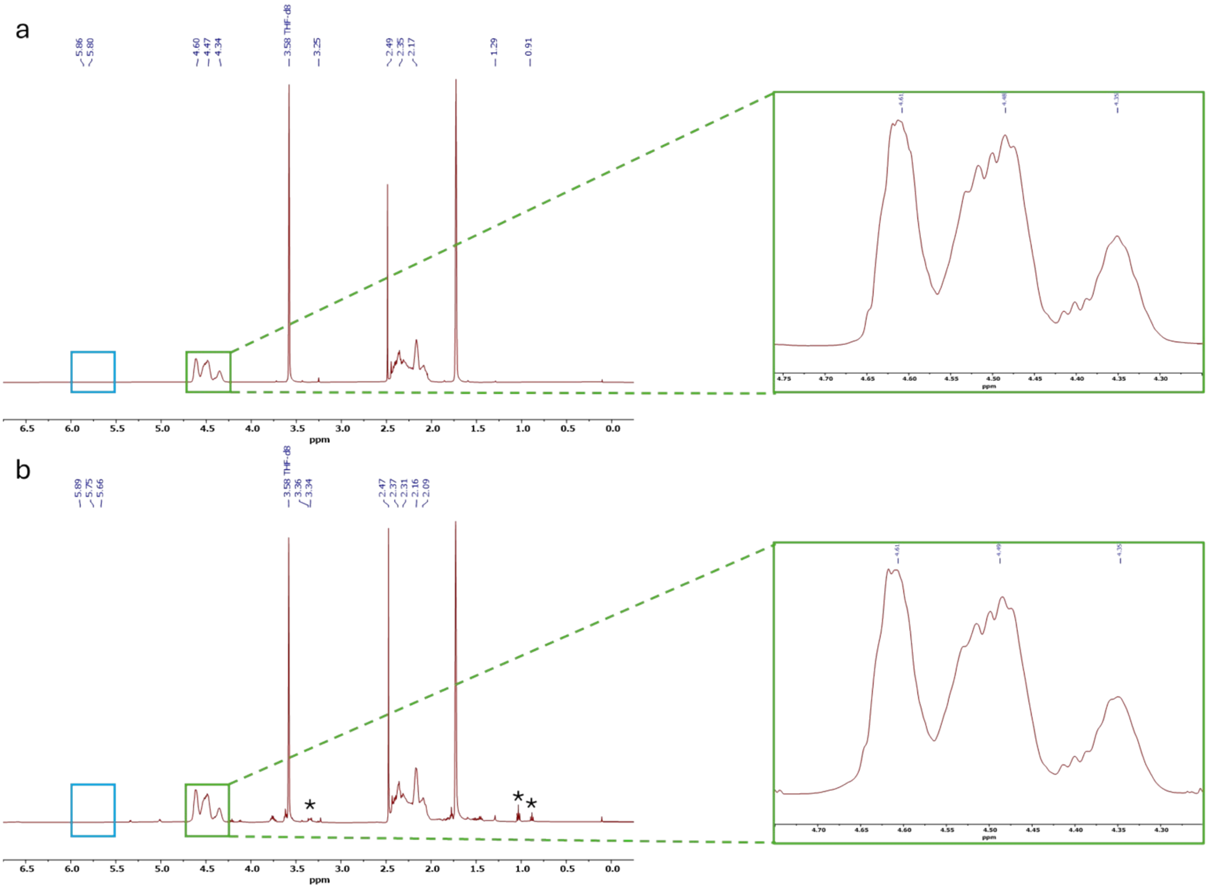

Supplementary Figure S1 – ^{1}H NMR spectra of a) PVC and b) PVC iodinated via phase-transfer catalysis for 24h. The remaining TBAB in b) is designated by asterisks (*).

b. Fourier Transform Infrared Spectroscopy

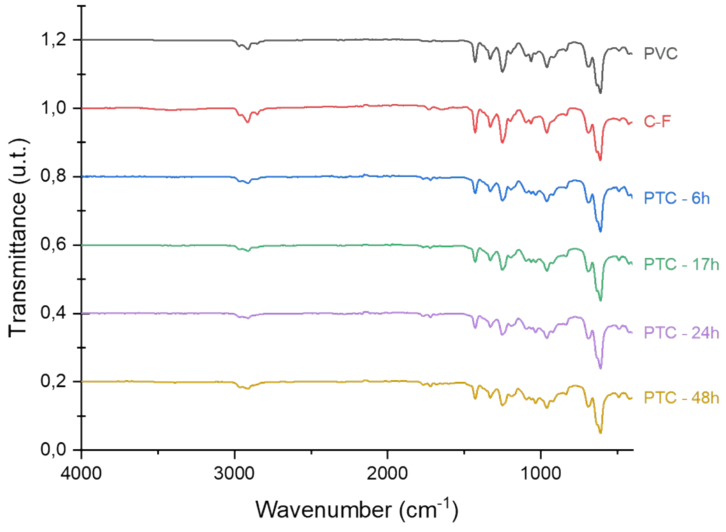

Supplementary Figure S2 – FT-IR of PVC and modified PVC samples iodinated via the Conant-Finkelstein reaction (C-F) or phase transfer catalysis (PTC) for 6, 17, 24, or 48 hours.

c. Thermogravimetric Analysis

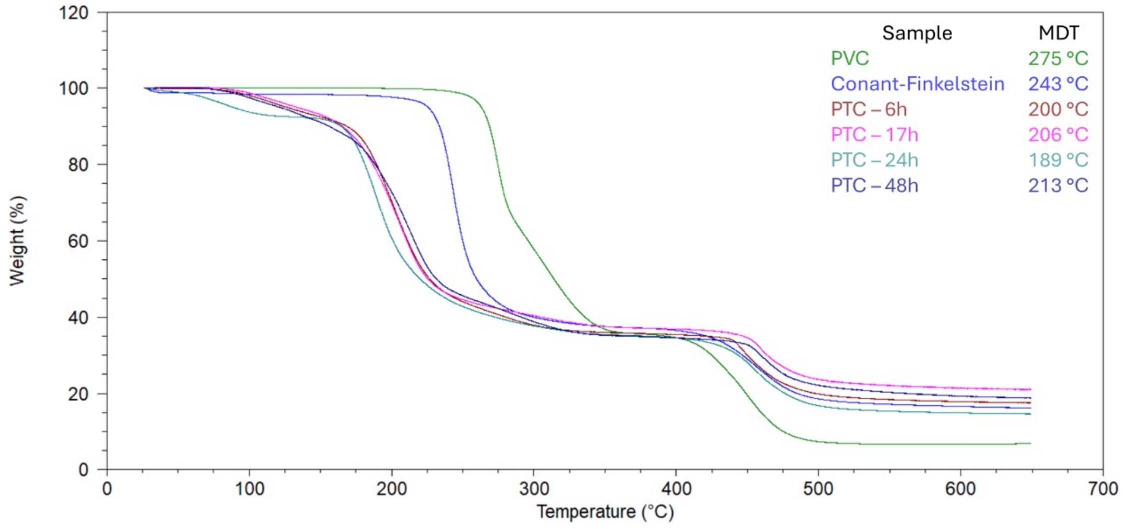

Supplementary Figure S3 – TGA of PVC and modified PVC samples iodinated via the Conant-Finkelstein reaction (C-F) or phase transfer catalysis (PTC) for 6, 17, 24, or 48 hours.

d. Size Exclusion Chromatography

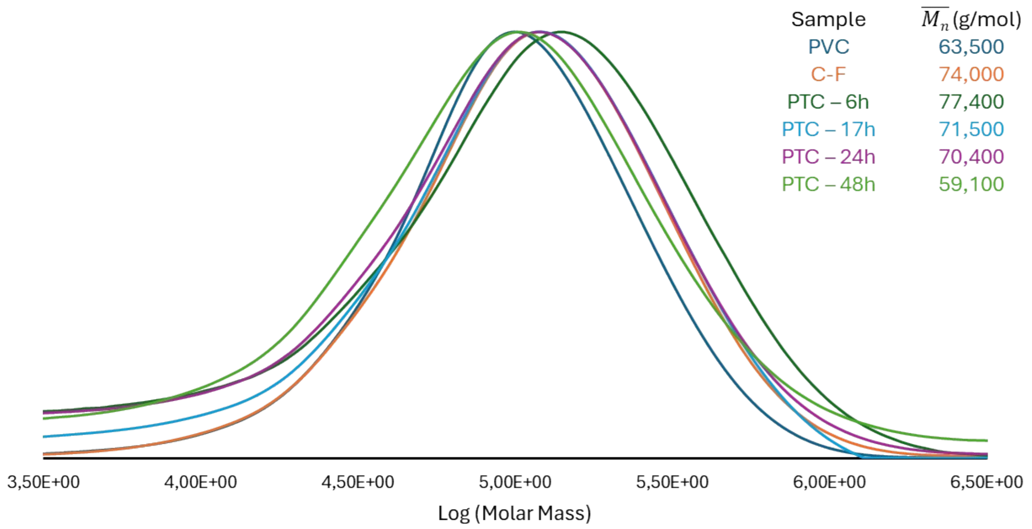

Supplementary Figure S4 – SEC traces of PVC and modified PVC samples iodinated via the Conant-Finkelstein reaction (C-F) or phase transfer catalysis (PTC) for 6, 17, 24, or 48 hours.

e. X-ray Photoelectron Spectroscopy

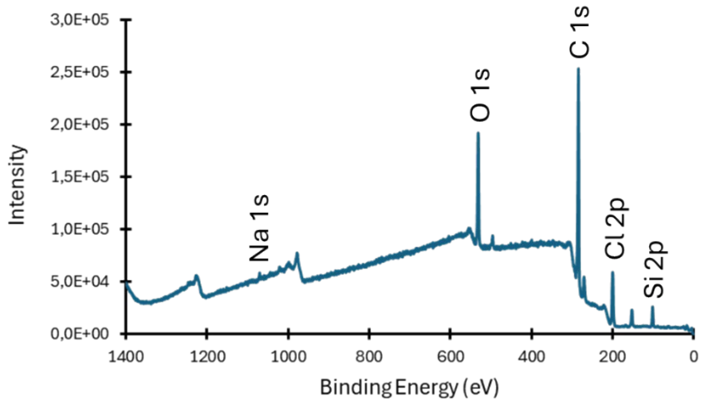

Supplementary Figure S5 – XPS spectrum of PVC.

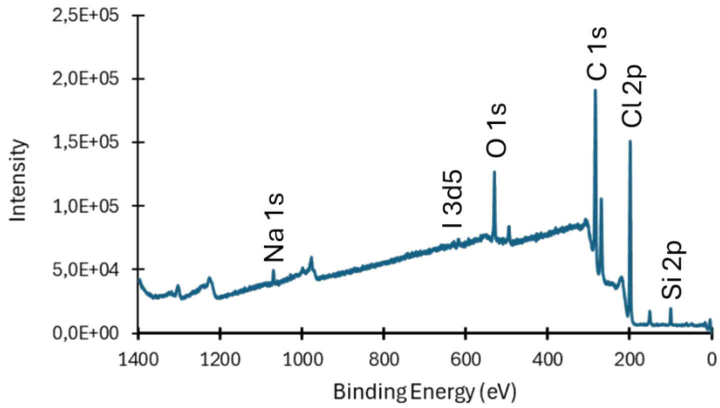

Supplementary Figure S6 – XPS spectrum of PVC iodinated via the Conant-Finkelstein reaction.

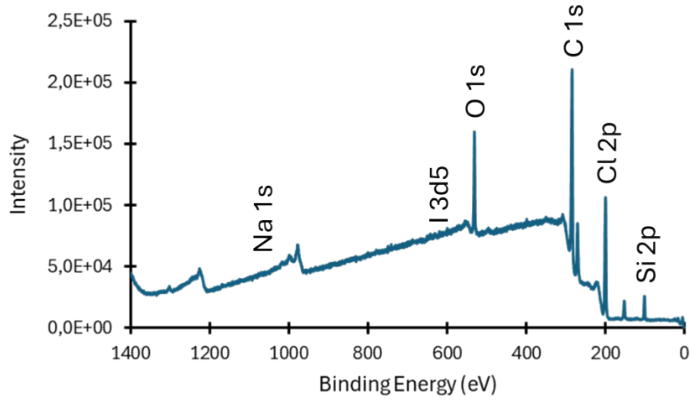

Supplementary Figure S7 – XPS spectrum of PVC iodinated via phase transfer catalysis for 6 hours.

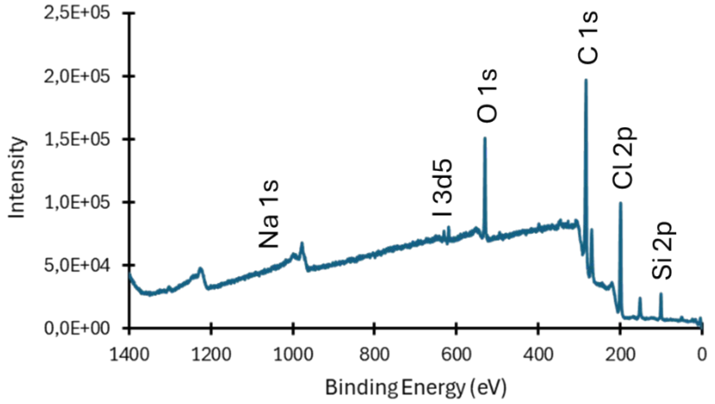

Supplementary Figure S8 – XPS spectrum of PVC iodinated via phase transfer catalysis for 17 hours.

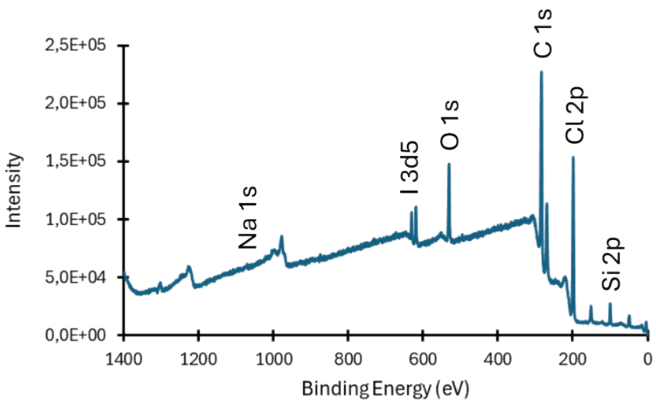

Supplementary Figure S9 – XPS spectrum of PVC iodinated via phase transfer catalysis for 24 hours.

f. Differential Scanning Calorimetry

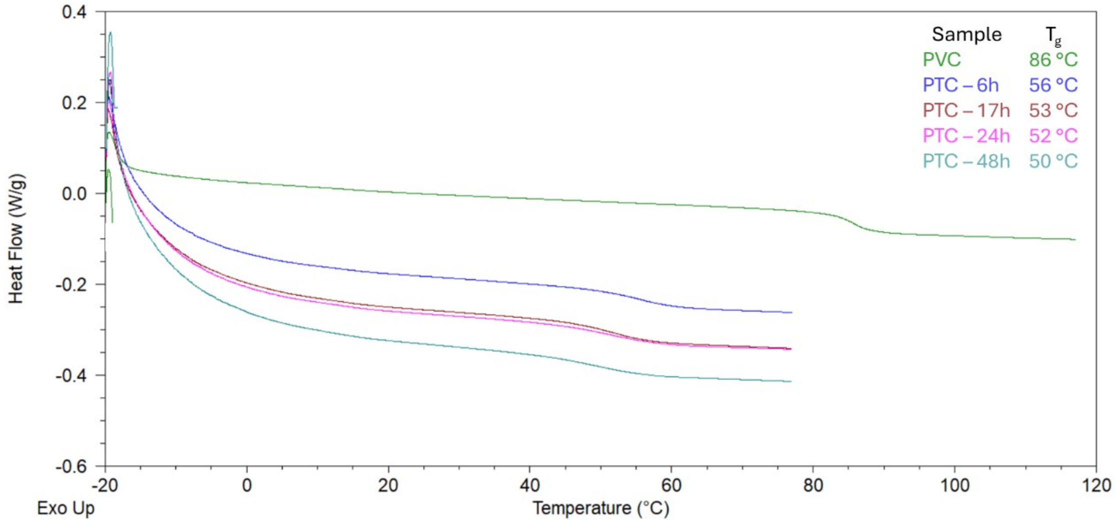

Supplementary Figure S10 – DSC of PVC and modified PVC samples iodinated via phase transfer catalysis (PTC) for 6, 17, 24, or 48 hours.

g. **Supplementary Table S1** – Summary of physicochemical and thermal properties of pristine and iodinated PVC samples.

	Method	*t (h)*	*MDT (°C)*	$\bar{M}_n$ *(g/mol)*	*C 1s (at%)*	*Cl 2p (at%)*	*I 3d5 (at%)*	*Na 1s (at%)*	T_g *(°C)*	*Contact angle (°)*
1	PVC	0	275	63,500	73.52	6.53	0	0.49	86	85.6 ± 1.7
2	C-F	24	243	74,000	67.30	19.23	0.10	1.52	/	/
3	PTC	6	200	77,400	69.78	12.48	0.05	0.25	56	86.8 ± 1.1
4	PTC	17	206	71,500	70.29	12.14	0.18	0	53	85.5 ± 2.3
5	PTC	24	189	70,400	69.87	15.79	0.48	0.16	52	85.9 ± 1.6
6	PTC	48	213	59,100	67.47	17.37	0.84	0.23	50	83.4 ± 4.3

2. CO_2BAM characterization

a. Nuclear Magnetic Resonance

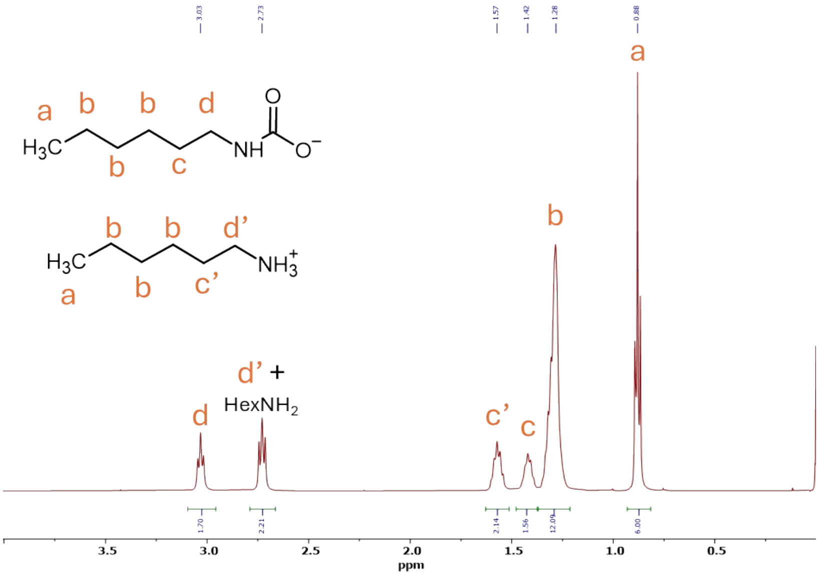

Supplementary Figure S11 – ^{1}H NMR spectrum of the amine only-based CO_2BAM.

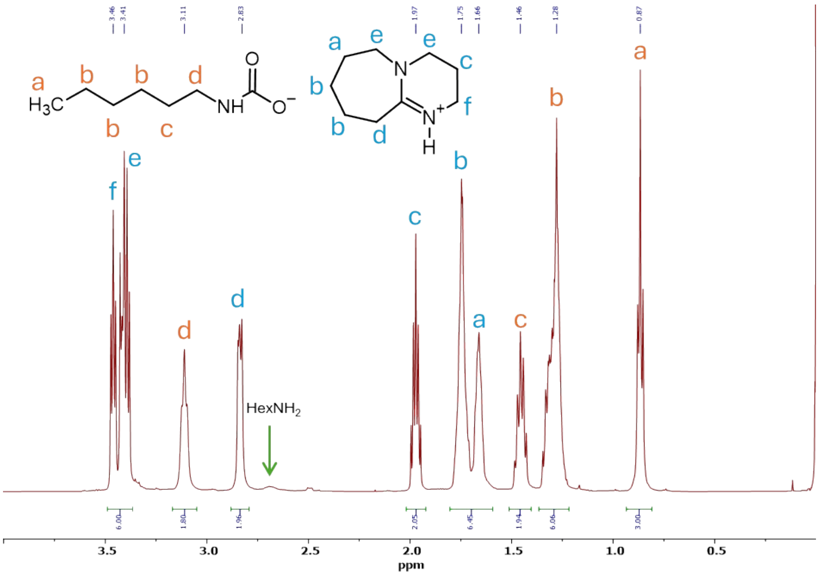

Supplementary Figure S12 – ^{1}H NMR spectrum of the hexylamine and DBU-based CO_2BAM.

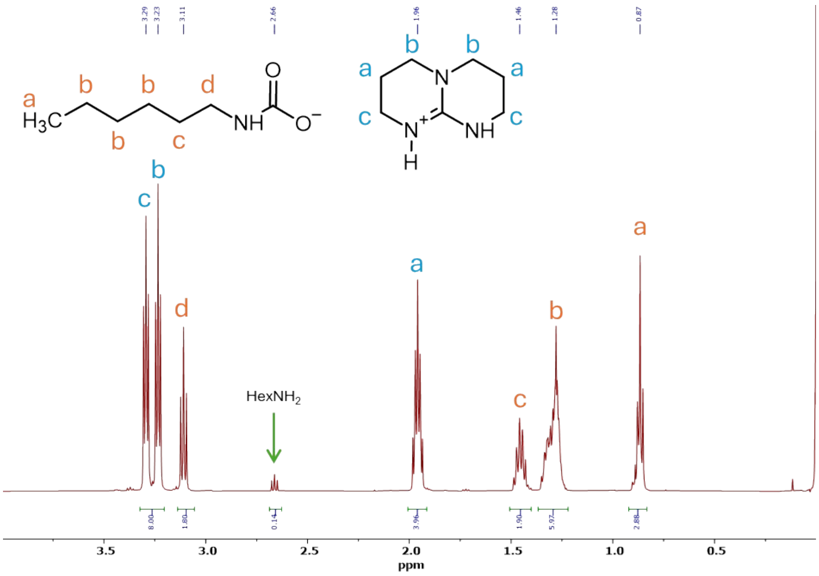

Supplementary Figure S13 – ^{1}H NMR spectrum of the hexylamine and TBD-based CO_2BAM.

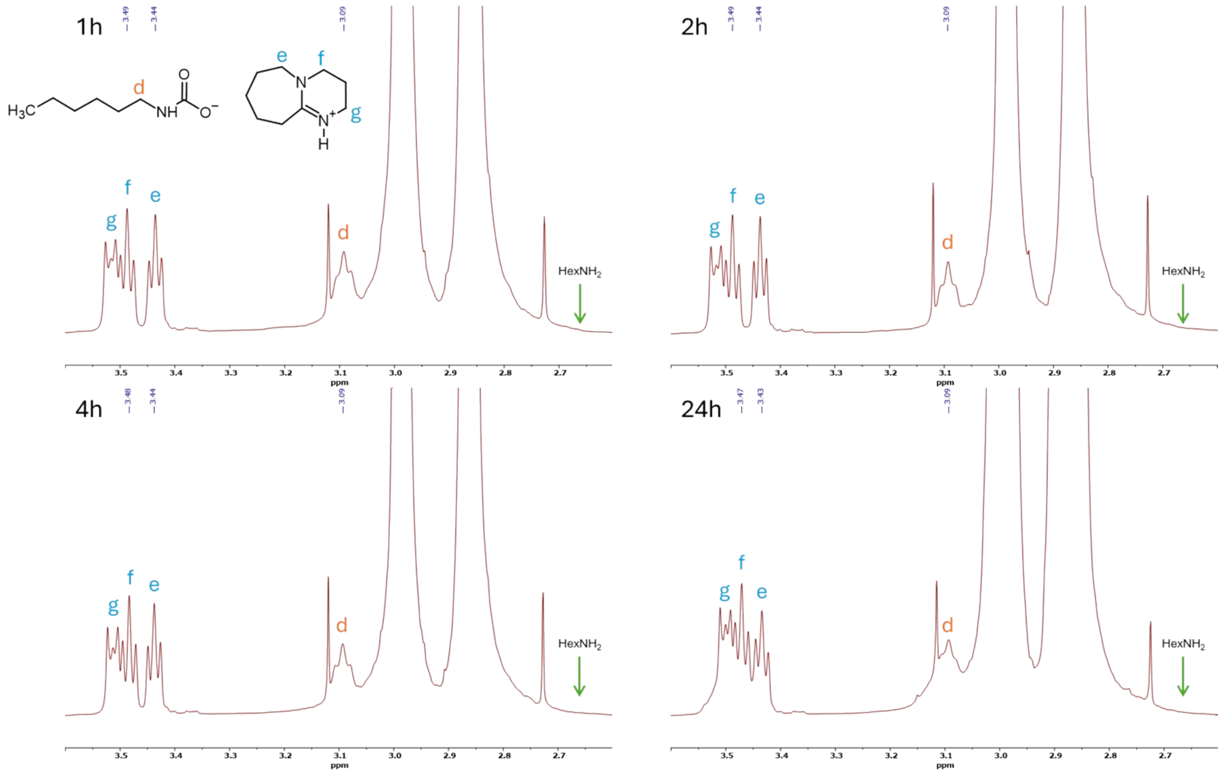

Supplementary Figure S14 – Stability of DBU-based CO_2BAM of 1-hexylamine in solution in DMF studied by ^{1}H NMR spectroscopy. The green arrow indicates where free hexylamine should appear in case of CO_2BAM degradation.

b. Thermogravimetric Analysis

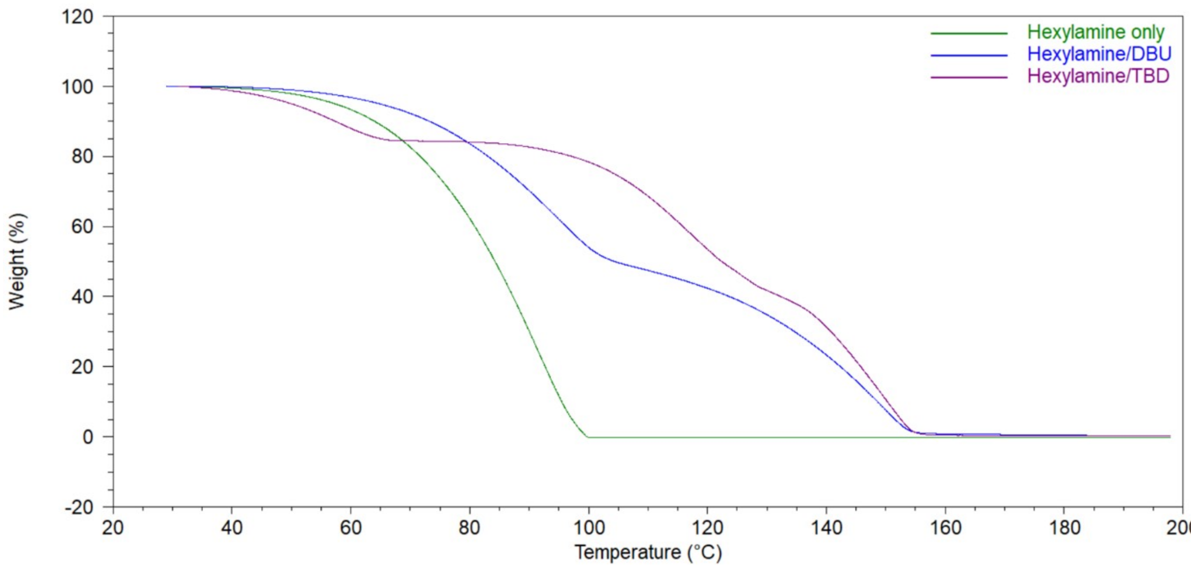

Supplementary Figure S15 – TGA thermograms of pristine 1-hexylamine, and hexylamine/DBU and hexylamine/TBD-based CO_2BAMs.

3. Reaction between iodinated PVC and DBU-based CO_2BAM – Product characterization

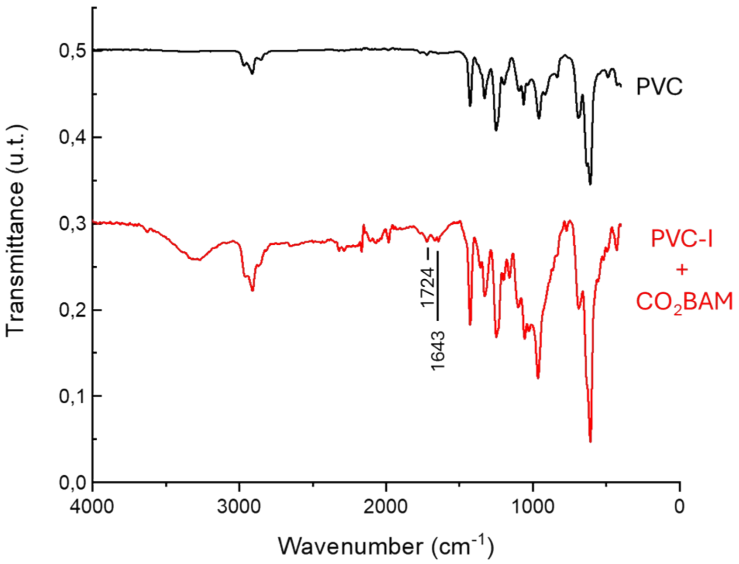

Supplementary Figure S16 – FT-IR spectra of pristine PVC and the product of reaction between PVC-I and the DBU-based CO_2BAM of 1-hexylamine.

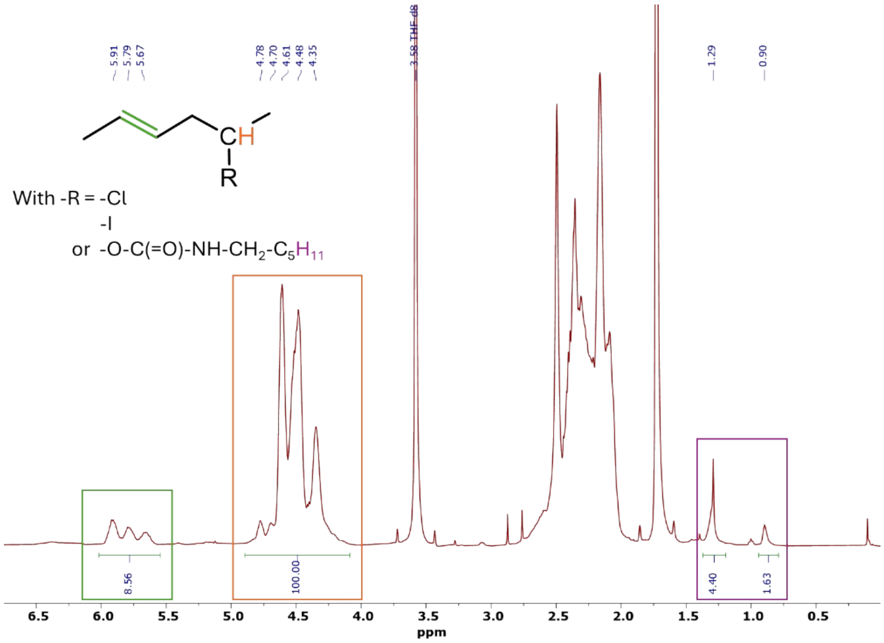

Supplementary Figure S17 - ^{1}H NMR spectrum of the reaction product between PVC-I and the DBU-based CO_2BAM of 1-hexylamine.
